# Validation of an Automated Fluorescence‐ and Image‐Based Viable Cell Counting Method for Fecal Microbiota Transplantation Drug Products

**DOI:** 10.1002/biot.70269

**Published:** 2026-06-17

**Authors:** Timon Lang, Fedja Farowski, Jozef Al‐Gousous, Peter Langguth, Maria J. G. T. Vehreschild

**Affiliations:** ^1^ Department II of Internal Medicine Goethe University Frankfurt University Hospital Frankfurt Infectious Diseases Frankfurt am Main Germany; ^2^ Department of Biopharmaceutics and Pharmaceutical Technology Johannes Gutenberg‐University Mainz Germany; ^3^ Department I of Internal Medicine University of Cologne Faculty of Medicine and University Hospital Cologne Cologne Germany; ^4^ German Centre for Infection Research (DZIF) Partner Site Bonn‐Cologne Cologne Germany; ^5^ Department of Pharmaceutical Sciences University of Michigan Ann Arbor USA

**Keywords:** analytical method validation, fecal microbiota transplantation, GMP, ICH‐Guideline Q2 (R2), viable bacterial quantification

## Abstract

Fecal microbiota transplantation (FMT) is an established treatment for recurrent *Clostridioides difficile* infection. As a drug product, FMT is subject to pharmaceutical quality standards, including accurate quantification of viable bacteria to ensure product consistency. We developed and validated an automated fluorescence‐ and image‐based viable‐cell counting method for FMT products; total‐cell enumeration was examined secondarily. The QUANTOM Tx system (Logos Biosystems) was validated according to ICH Q2(R2) using *Escherichia coli* and *Bacillus subtilis* as reference strains. The Viable Cell Staining Kit was validated with *Escherichia coli*, whereas the Total Cell Staining Kit was examined in selected experiments using both strains. Applicability to complex FMT matrices was confirmed using authentic fecal samples. Viable‐cell counting showed strong linearity across a range of 9.4 × 10^6^–1.2 × 10^8^ cells/mL, with adequate accuracy and precision. Specificity was ensured by particle‐size gating, unaffected by nonviable cells or diluent. Robustness was confirmed across operators and time points. Fecal samples showed linear quantifiability and acceptable precision; total‐cell enumeration fulfilled linearity and precision requirements. The validated method enables rapid, reproducible viable‐cell quantification suitable for Quality Control of FMT drug products, supporting formulation development, and dosing verification. Viable‐cell quantification remains the analytical focus, despite limitations in spore detection. Total‐cell enumeration provides complementary process information.

## Graphical Abstract and Lay Summary

This study presents the first systematic development and analytical validation of an automated fluorescence‐ and image‐based viable‐cell counting method for fecal microbiota transplantation (FMT) drug products in accordance with ICH Q2(R2). The validated QUANTOM Tx approach enables rapid and reproducible quantification of enzymatically active bacteria in complex fecal matrices independent of culturability, supporting standardized quality control and batch comparability.

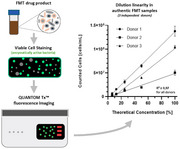

## 1 Introduction

Fecal microbiota transplantation (FMT) has become a standard of care in the treatment of recurrent *Clostridioides difficile* infections (CDI), where conventional antibiotic therapy often fails. In recent years, FMT has also gained attention as a potential treatment for a range of gastrointestinal and even extraintestinal diseases, including inflammatory bowel disease (IBD), irritable bowel syndrome (IBS), and metabolic diseases [1]. The underlying therapeutic concept relies on the restoration of a diverse and balanced gut microbiota, increasingly recognized as a key determinant of both intestinal and systemic health [2].

According to the German Federal Institute for Drugs and Medical Devices (BfArM) [3], FMT is classified as a drug product and is therefore subject to strict GMP guidelines and the German Medicines Act (AMG). At European level, FMT has been included in the new Regulation on Substances of Human Origin (SoHO), which took effect in August, 2024. A transitional period has been established, during which the SoHO framework for intestinal microbiota will be implemented in a stepwise manner, with mandatory requirements applying from 2027, while the specifics of national implementation remain under deliberation. Outside Europe, regulatory frameworks for FMT remain heterogeneous. In the United States, the Food and Drug Administration (FDA) classifies fecal microbiota products intended for the diagnosis, prevention, treatment, or cure of disease as biological products, which are subject to the regulatory oversight of the Center for Biologics Evaluation and Research (CBER) [4]. In China, regulatory frameworks for FMT and microbiota‐based therapies have recently evolved through the implementation of the Regulation on the Administration of Clinical Research and Clinical Translational Application of New Biomedical Technologies, involving both the National Health Commission (NHC) and the National Medical Products Administration (NMPA) [5]. Regulatory interpretations further describe a dual‐track framework for microbial therapies and suggest that certain microbiota transplantation approaches are regulated separately from the category of new biomedical technologies [6]. Expert consensus documents additionally emphasize the need for rigorous donor screening, standardized manufacturing processes, enhanced quality control and product standardization for FMT products [7, 8]. Consequently, pharmaceutical quality requirements, including GMP‐based controls, continue to apply at present. In addition to stringent donor screening protocols, a key aspect of FMT product consistency is the accurate quantification of the bacterial load, especially of viable, enzymatically active cells, which are hypothesized to be the primary drivers of the therapeutic effect [9].

To date, diversity profiling of bacterial communities in FMT products has been successfully established using molecular methods such as 16S rRNA gene sequencing. However, robust, quantitative, and standardized methods for assessing viable bacterial cell counts in FMT products are still lacking. Conventional culture‐based methods are time‐consuming and insufficient to capture non‐culturable species. Although advanced techniques such as fluorescence‐activated cell sorting (FACS) or propidium monoazide‐based sequencing (PMA‐Seq) can provide information on the viable fraction of the bacteria, these techniques require specialized and expensive instruments. This highlights the need for rapid, reliable, and reproducible alternatives suitable for routine QC.

In this context, the QUANTOM Tx cell counter (Logos Biosystems, South Korea) was chosen as the analytical instrument. This automated and image‐based system employs fluorescence staining and software‐driven image analysis to differentiate and enumerate marked viable and total bacterial cells.

The Viable Cell Staining Kit relies on calcein‐acetoxymethyl ester (calcein‐AM), a nonfluorescent, lipophilic dye that is able to pass through bacterial membranes. Once inside the cell, ubiquitous intracellular esterases cleave the acetoxymethyl groups, generating calcein, a hydrophilic, highly fluorescent anion (excitation/emission: 496/520 nm) that is retained in cells with intact membranes; the resulting intracellular fluorescence serves as a proxy for metabolic viability [10, 11].

As demonstrated in previous studies, the method has potential for use in microbial applications [12, 13]. However, it is still necessary to formally validate the method's applicability to microbial enumeration from complex matrices, such as FMT. Specifically, the ability to selectively quantify viable, enzymatically active bacteria in the presence of dead cells and other potentially interfering components of the FMT matrix has not been systematically evaluated. This study therefore validated the QUANTOM Tx system according to ICH Guideline Q2(R2) [14], focusing on viable‐cell quantification with the Viable Cell Staining Kit and complementary assessment of total‐cell enumeration. Therefore, *Escherichia coli* (*E. coli*) and *Bacillus subtilis* (*B. subtilis*) were specifically selected as model organisms representing distinct bacterial morphologies and cell wall structures (rod‐shaped Gram‐negative and rod‐shaped Gram‐positive bacteria), as well as different physiological characteristics, including vegetative and spore‐forming states. In addition, both organisms are widely established and well‐characterized reference strains suitable for analytical method validation. This selection enables a comprehensive assessment of the method's applicability and robustness across diverse bacterial characteristics relevant to fecal microbiota analysis.

By systematically examining specificity, linearity, accuracy, precision, and robustness—including the assessment of authentic fecal samples—this study provides a comprehensive analytical validation of the QUANTOM Tx method and contributes to the standardization and enhancement of QC procedures for FMT products in clinical and research applications.

## 2 Materials and Methods

All validation procedures were conducted according to ICH Guideline Q2(R2) [14] and a predefined internal validation protocol detailing methodological parameters, acceptance criteria, and procedures.

### 2.1 Materials

The QUANTOM Tx Cell Counter (Logos Biosystems, South Korea) served as the primary analytical device. Two fluorescence‐based cell staining kits were applied: the QUANTOM Viable Cell Staining Kit (Q13502) and the QUANTOM Total Cell Staining Kit (Q13501). The Viable Cell Staining Dye (Q13201) was diluted with dimethyl sulfoxide (DMSO; Q13003) in accordance with the manufacturer's instructions. For total cell staining, QUANTOM Total Cell Staining Dye (Q13101) was applied in combination with the Total Cell Staining Enhancer (Q13002) to improve membrane permeability.

Certified quantitative reference standards were used: *E. coli* (BioBall E. COLI NCTC 12923 10E8) and *B. subtilis* (BioBall B. subtilis NBRC 13722 10E8), both from bioMérieux (Nürtingen, Germany). BioBall reference concentrations are expressed as colony forming units (CFU)/mL according to the certificate of analysis, whereas QUANTOM Tx results are reported as cells/mL, reflecting the instrument output of fluorescence‐based cell enumeration. BioBalls were rehydrated with 1.1 mL BIOBALL Rehydration Fluid (cat. no. 56021) to yield suspensions of ≈1 × 10^8^ CFU/mL.

Additional reagents included sterile 0.9% sodium chloride (NaCl) solution (B. Braun Melsungen AG, Germany) for dilutions and 96% ethanol (EMPROVE EXPERT, Merck, Darmstadt, Germany) for spore preparation. Sample incubation and heat treatment were performed in an Eppendorf ThermoBlock (Eppendorf AG, Hamburg, Germany). QUANTOM Cell Loading Buffer I (Q13001) served to ensure homogeneous cell distribution in QUANTOM M50 Cell Counting Slides (Q12001). Each chamber was loaded with 5.5 µL sample mixture and centrifuged at 400 × *g* for 10 min using the QUANTOM Centrifuge (Logos Biosystems, South Korea). Furthermore, processed FMT suspensions (fecal slurry) from three individual donors were included to assess method applicability in complex biological matrices.

## 3 Methods

### 3.1 Preliminary Experiments

Preliminary studies were performed to optimize procedural settings and evaluate sample stability. To assess the impact of procedural timing on bacterial viability, *E. coli* suspensions containing a defined number of enzymatically active cells were exposed to four handling conditions: immediate processing, a 2‐h delay on dry ice, a 2‐h delay at room temperature prior to staining (pre‐staining holding time), and a 2‐h delay at room temperature following the staining procedure (post‐staining stability).

Blank matrices (sterile 0.9% NaCl and Rehydration Fluid) were analyzed to determine background fluorescence, while heat‐treated fecal slurry was tested to evaluate potential nonspecific staining in a complex biological matrix. Based on these results, detection thresholds were adjusted to minimize false‐positive signals. Additionally, ethanol‐treated *B. subtilis* suspensions were analyzed to assess detection of spores, which were expected to show limited response in viability staining due to low metabolic activity.

### 3.2 Cell Counting Parameters

Detection settings were optimized in preliminary experiments and adapted according to manufacturer's recommendations [15], a published protocol [16] and matrix‐specific requirements. For viability assays, the particle size range was set to 1.4–6 µm for the *E. coli* reference strain and to 1–6 µm for FMT samples to account for their heterogeneous composition and smaller bacterial species. The roundness threshold was set to 30%, de‐clustering to level 2, and LED intensity fixed at level 9, with LED intensity adjustments restricted to a narrow operational range around the default setting to accommodate sample‐specific fluorescence characteristics.

For total‐cell counts, counting parameters were set to a particle size range of 1.4–9 µm. The lower threshold was selected based on preliminary experiments indicating that signals below this size predominantly originated from non‐cellular background. Roundness was fixed at 30% for *E. coli*, while no roundness threshold was applied for *B. subtilis* to account for spore morphology. De‐clustering was set to level 7, and LED intensity to level 5. Unless otherwise specified, each sample was applied to both chambers of a QUANTOM M50 Cell Counting Slide. Values from both chambers were averaged to yield a single data point per replicate.

### 3.3 Specificity and Selectivity

Specificity was assessed using blank matrices, heat‐treated fecal slurry, and mixtures of viable and heat‐inactivated *E*. *coli*. Heat inactivation was performed by incubating samples at 95°C for 1 h. Test suspensions represented 90%, 40%, and 20% viable cells, prepared by volumetric mixing with either heat‐killed *E. coli* suspension or 0.9 % NaCl as diluent. The latter served as reference data and was generated during the external validation. Each condition was analyzed in triplicate with independently prepared samples.

### 3.4 Linearity and Range

Linearity was evaluated across seven serial 1:2 dilutions of BioBall suspensions. The Viable Cell Staining Kit (*E. coli)* covered 1.8 × 10^6^ to 1.2 × 10^8^ CFU/mL; total‐cell counts (*E. coli* and *B. subtilis*) covered 1.6 × 10^6^ to 1.1 × 10^8^ cells/mL. Each dilution was measured in triplicate, with one QUANTOM M50 slide per replicate.

For FMT samples, due to the lack of a certified reference standard and the absence of a true reference value for absolute bacterial quantification of enzymatically active bacterial cells in complex native fecal matrices, linearity was assessed as dilution linearity to demonstrate proportionality. The bacterial concentration of an initial 1:480 dilution was defined as nominal 100% reference value. Subsequent serial 1:2 dilutions (down to 6.25%) were prepared and theoretical values were calculated relative to this internal reference. Measured concentrations were plotted against these relative theoretical values, and linear regression was used to calculate slope, intercept, and coefficient of determination (*R* ^2^). Relative deviation (RD%) between measured and calculated relative values was determined, with a predefined acceptance criterion of ± 20%. The LLOQ was calculated according to ICH Q2(R2) [14] using residual standard deviation (s) and the slope of the regression line (S), applying the formula LLOQ = 10·s/S.

### 3.5 Accuracy

Accuracy was assessed using the Viable Cell Staining Kit with *E. coli* BioBall suspensions. The data sets from linearity experiments were applied for this evaluation. Measured concentrations were compared to theoretical values derived from the lot‐specific colony forming units (CFU) content in the BioBall certificate of analysis. Seven dilution levels (1.8 × 10^6^ to 1.2 × 10^8^ CFU/mL) were analyzed in triplicate on three independent days. Accuracy was expressed as recovery rate (A%), calculated as the ratio measured to theoretical concentration, with a predefined acceptance range of 85%–115%, accounting for the ± 5% variation specified by the manufacturer.

To confirm robustness and broader applicability, an independent validation set was analyzed for both the Viable and Total Cell Staining Kits using *E. coli* BioBall suspensions at three additional concentrations (20%, 40%, and 90% relative to the nominal 1 × 10^8^ CFU/mL stock). For total‐cell staining, the same concentrations were also tested with *B. subtilis* BioBall suspensions. Each concentration was independently prepared in triplicate and analyzed as previously described. Recovery (A%) and relative deviation (RD%) were calculated against expected values derived from the respective regression models. Acceptance criteria were accuracy of 85%–115% recovery and precision with CV ≤ 10%. The external dataset provided further confirmation of the suitability of both assays for routine application.

### 3.6 Precision

Precision was examined for both the Viable Cell Staining Kit and the Total Cell Staining Kit using *E. coli* and *B. subtilis* reference standards. Repeatability (intra‐day precision) was determined by analyzing six independently prepared suspensions (≈1 × 10^8^ CFU/mL) on the same day by one operator. Intermediate precision included inter‐day (assessed across 2 days) and inter‐operator comparisons (two operators on day 2), with six freshly prepared suspensions per condition. Each replicate was measured on a separate QUANTOM M50 slide. Precision was expressed as coefficient of variation (CV%) of averaged replicate values, with a predefined acceptance threshold of CV values <10% for both kits.

For authentic fecal samples, precision was evaluated within the linearity experiments by calculating CV% for each dilution. In the absence of predefined acceptance limits, a threshold of 15% was considered reasonable, reflecting established guidelines for complex biological matrices and accounting for the higher variability inherent to fecal‐based samples [17].

### 3.7 Robustness

Robustness was evaluated indirectly through inter‐day and inter‐operator precision experiments and supported by selected preliminary studies addressing procedural variables such as timing effects. For the latter, the same BioBall dilution level was used across all conditions, enabling direct within‐experiment comparison of timing effects. These elements were considered sufficient to demonstrate the method´s reliability under typical routine conditions.

### 3.8 Statistical Analysis

Statistical analyses were carried out using GraphPad Prism Version 8.0.1 (GraphPad Software, San Diego, CA, USA) and Microsoft Excel (Version 2501). For each validation parameter, descriptive and statistical metrics were calculated. Mean values and standard deviations were determined as applicable, and precision was expressed as coefficient of variation (CV%) across replicate measurements. Linearity was evaluated by simple linear regression, including slope, intercept, and coefficient of determination (*R^2^*). Residual plots were reviewed to confirm model fit and assess homoscedasticity. Acceptance criteria followed ICH Q2(R2) [14], with additional adjustments considered for complex biological matrices.

## 4 Results

The specificity and selectivity of the QUANTOM Tx Viable Cell Staining Kit were evaluated using blank matrices (BIOBALL Rehydration Fluid and sterile 0.9% NaCl). Low‐level signals from non‐cellular particles (≈1.0–1.1 µm) were effectively excluded by setting the minimum particle size threshold to 1.4 µm for *E. coli*, ensuring specific detection of viable bacterial cells. For authentic fecal samples, the threshold was reduced to 1.0 µm to accommodate smaller or atypically shaped bacterial species present in the fecal matrix. This adjustment proved acceptable, as heat‐treated fecal suspensions, containing no viable cells, showed no detectable fluorescence above the threshold; if noncellular particles with intrinsic autofluorescence were present, they would be expected to remain visible under these conditions. Thus, inclusion of a broader range of morphologically diverse bacteria was enabled without substantially compromising analytical specificity.

**FIGURE 1:**
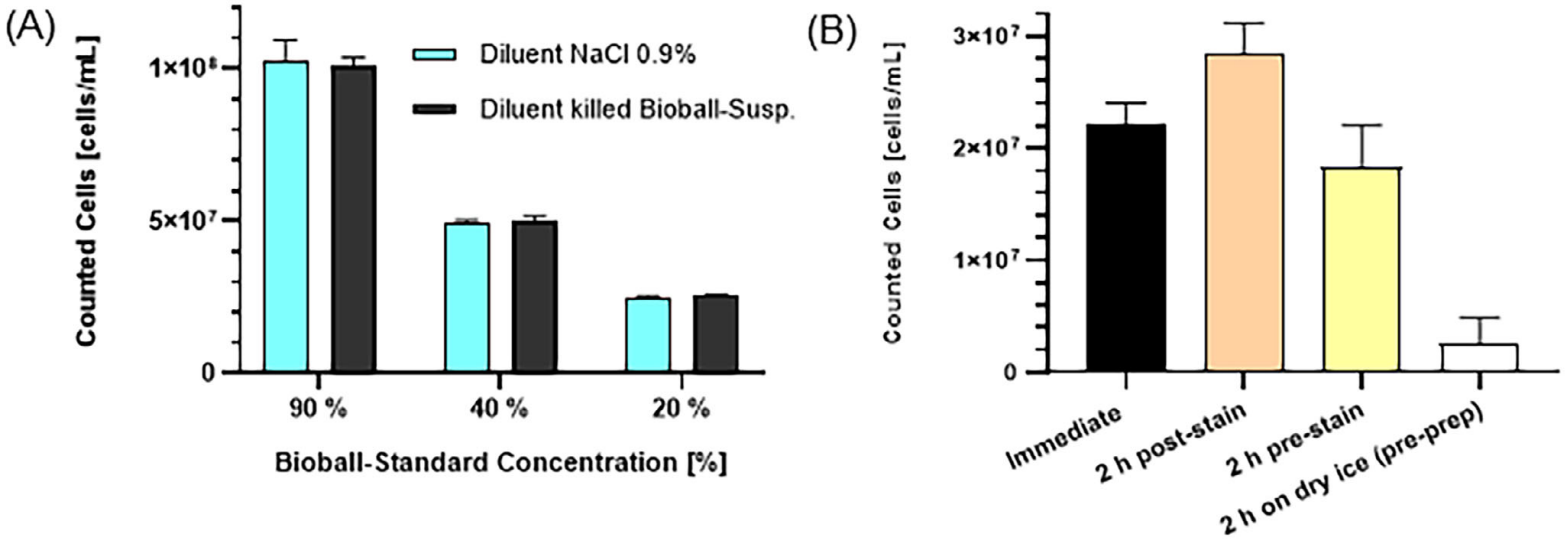
Selectivity and robustness of the QUANTOM Tx Viable Cell Staining Kit. (A) *E. coli* at 90%, 40%, and 20% viability in 0.9% NaCl or heat‐inactivated BioBall diluent. (B) Timing‐effects on viable counts: immediate readout, 2 h post‐stain, 2 h pre‐stain, and 2 h on dry ice (pre‐prep).

To evaluate potential interference from non‐viable cells, *E. coli* suspensions with defined proportions of viable bacteria (90%, 40%, 20%) were prepared by mixing viable cells with heat‐inactivated *E. coli* (95°C, 1 h). These were compared to equivalent dilutions using 0.9% NaCl as diluent. All conditions were run in triplicate. Statistical analysis using an ordinary two‐way ANOVA revealed a highly significant effect of the defined viability levels on measured viable‐cell counts (*p * <  0.0001). In contrast, diluent type had no significant effect (*p *= 0.9545), and no interaction between viability level and diluent was observed (*p *= 0.7919). The mean difference between NaCl and heat‐inactivated cell dilutions was −8.9 × 10^4^ cells/mL, corresponding to <0.2% of the measured values. These results indicate that the presence of nonviable cells did not interfere with viability quantification (Figure 1A).

Robustness of viable‐cell measurements was assessed by inter‐day and inter‐operator precision (see Section 2.2.6) and procedural timing experiments. Statistical differences between timing conditions were evaluated using ordinary one‐way ANOVA followed by Tukey's multiple comparison test. No significant differences were observed between immediate measurement and a 2‐h delay prior to staining (*p *> 0.05). In contrast, a 2‐h delay post‐staining resulted in significantly increased viable‐cell counts compared to both other conditions (*p * <  0.05). Furthermore, keeping cryoprotectant‐free aliquots on dry ice resulted in a decrease in viable cell numbers compared to samples processed at room temperature (Figure 1B).

**FIGURE 2:**
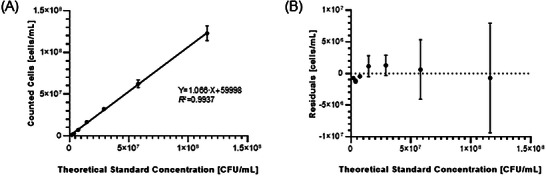
(A) Linear regression of serial dilutions of the reference strain *E. coli* using the Viable Cell Staining Kit. (B) Residual plot of the linear regression, illustrating the distribution and magnitude of residuals across the tested concentrations.

Linearity was assessed using seven serial 1:2 dilutions of *E. coli* BioBall suspensions (covered concentration ≈1.2 × 10^8^ to 1.8 × 10^6^ CFU/mL), analyzed over 3 independent days. For the Viable Cell Staining Kit, linear regression yielded the equation Y = 1.066·X + 59998 with a coefficient of determination *R* ^2^ = 0.9937 (*p* < 0.0001) (Figure 2A). RD% remained within ± 20% down to 7.24 × 10^6^ cells/mL. At lower concentrations (3.62 × 10^6^ and 1.81 × 10^6^ CFU/mL), deviations increased to −30.6% and −36.9%, respectively.

LLOQ was determined as 9.44 × 10^6^ cells/mL according to ICH Q2(R2) criteria [14]. Accuracy and precision were acceptable above this threshold. The mean absolute residual was 8.75 × 10^5^ cells/mL with a standard deviation of 1.01 × 10^6^ cells/mL. Visual inspection of the residual plot (Figure 2B) indicating good agreement between observed and predicted values without systematic deviation or heteroscedasticity.

For the Total Cell Staining Kit, linearity was assessed for both *E. coli* and *B. subtilis* over the same dilution range. Linear regression for *E. coli* yielded *Y* = 1.09 × 10^6^·X + 2.24 × 10^6^ (*R* ^2^ = 0.9935; *p* < 0.0001) and for *B. subtilis Y* = 1.38 × 10^6^·X + 1.18 × 10^6^ (*R* ^2^ = 0.9962; *p* < 0.0001). Relative deviations remained within ±20% down to 5.03 × 10^6^ cells/mL for *E. coli* and 5.04 × 10^6^ cells/mL for *B. subtilis;* below these levels, deviations exceeded acceptance criteria. Residual analysis showed homogeneous distribution in both cases. LLOQs were calculated at 1.36 × 10^7^ cells/mL for *E. coli* and 9.17 × 10^6^ cells/mL for *B. subtilis*.

**FIGURE 3:**
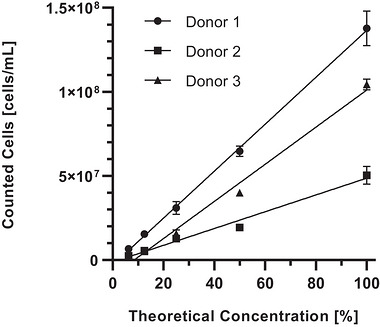
Linear regression of serial dilutions of three processed FMT suspensions derived from different donors using the Viable Cell Staining Kit.

Linearity in authentic matrix was confirmed by analyzing three independent fecal slurries from different donors at five dilution levels (100% to 6.25%). An initial 1:480 dilution was defined as the starting point (100%) to keep concentrations within the validated range. Linear regression yielded *R* ^2^ values of 0.9917, 0.9698, and 0.9882 (all *p* < 0.0001), confirming a strong linear correlation between defined dilution levels and measured bacterial counts (Figure 3).

Accuracy of the Viable Cell Staining Kit was assessed using *E. coli* BioBall suspensions at seven concentration levels ranging from 1.8 × 10^6^ to 1.2 × 10^8^ CFU/mL. Recovery rates (A%) were calculated based on lot‐specific CFU values. For concentrations ≥7.24 × 10^6^ CFU/mL, recovery ranged from 101.4% to 114.8%, falling within the acceptance range of 85%–115%. At lower concentrations (3.62 × 10^6^ and 1.81 × 10^6^ CFU/mL), recovery dropped to 75.1% and 69.3%, respectively. All concentrations meeting accuracy criteria were above the defined LLOQ, supporting the calculated quantification limit.

External validation was conducted for both staining kits at three concentrations (90%, 40%, 20%). For the Viable Cell Staining Kit, *E. coli* data with recovery rates from 98.0% to 107.6% and relative deviations between −8.1% and 0.6%, confirming excellent performance.

For the Total Cell Staining Kit recovery accuracy for *B. subtilis* met the ±20% deviation limit at all tested levels (RD%: 5.7%–13.7%), *E. coli* samples in this independent set exceeded this limit across all concentrations (RD%: 23.1%–37.0%), suggesting potential variability in total cell staining efficiency for *E. coli* under these specific conditions, possibly reflecting differences between spore based and vegetative BioBall materials.

Precision was evaluated for both kits using undiluted BioBall suspensions (≈1 × 10^8^ CFU/mL).

Repeatability (intra‐day) was assessed by analyzing six independently prepared replicates per day. For the Viable Cell Staining Kit, CVs ranged from 2.3% to 3.8% across two operators. For the Total Cell Staining Kit, intra‐day CV ranged from 3.2% to 6.4% for *E. coli*, and 2.3% to 4.0% for *B. subtilis*, confirming acceptable repeatability.

Intermediate precision was assessed by evaluating inter‐day and inter‐operator variation. For the Viable Cell Staining Kit, inter‐day CV (*E. coli*) was 1.3%, inter‐operator CV was 2.9%, both well below the <10% threshold. For the Total Cell Staining Kit, inter‐day CV for *E. coli* reached 10.4%, slightly exceeding the threshold, for *B. subtilis*, inter‐day CV remained low at 1.0%. Inter‐operator CVs were 0.2% (*E. coli*) and 1.3% (*B. subtilis*).

Except for the single inter‐day CV for *E. coli* Total Cell counts, all results met the acceptance criteria, demonstrating robust method reproducibility.

Precision for fecal samples was evaluated by calculating the coefficient of variation (CV%) across replicates at five dilution levels (100% to 6.25%). CVs were ≤10% for nearly all dilutions. Slightly elevated CVs were observed at lower concentrations (12.4% and 14.3% for Donor 1 and Donor 3 at 25% dilution) and at the 100% level for Donor 2 (10.6%). Given the inherent biological variability of fecal microbiota, the acceptance criteria from the ICH M10 guideline for bioanalytical method validation [17] (CV ≤ 15%) provided a scientifically suitable threshold. All CVs remained within this 15% limit, confirming the method's suitability for heterogeneous bacterial suspensions such as FMT preparations.

## 5 Discussion

The QUANTOM Tx Viable Cell Staining Kit was successfully validated as a specific, linear, and precise method for enumerating viable bacteria in both standardized reference suspensions and complex FMT matrices. Specificity testing demonstrated that setting a minimum particle size threshold of 1.4 µm for *E. coli* and 1.0 µm for FMT samples effectively excluded non‐cellular debris while preserving the detection of smaller or atypically shaped bacteria, reflecting the characteristic size distribution of bacterial cells relative to matrix‐derived background particles. Furthermore, roundness (30%) and declustering (level 2) parameters proved optimal for the robust quantification of enzymatically active cells across various morphologies, supporting applicability to different preparations based on fecal matrices.

Linearity testing with BioBall *E. coli* reference standards established a validated working range from 9.44 × 10^6^ to 1.2 × 10^8^ cells/mL, within which accuracy and precision consistently met ICH Q2(R2) criteria [14]. The LLOQ, derived from regression analysis and confirmed by reduced recovery and precision below 9.44 × 10^6^ cells/mL, defines the lowest concentration at which reliable live‐cell quantification can be achieved using this validated method. External validation using independently prepared *E. coli* suspensions confirmed the method's accuracy and robustness under realistic laboratory conditions.

Authentic fecal samples were analyzed starting from a 1:480 dilution (≈1.2 × 10^8^ viable cells/mL) and serially diluted to cover a biologically relevant concentration range. This approach confirmed strong linearity (*R* ^2^ ≥ 0.9698) and acceptable precision under matrix conditions. CV values remained ≤10% at most dilutions and ≤15% even at the lowest levels. This aligns with the FDA Bioanalytical Method Validation Guidance and ICH M10 for bioanalytical method validation in complex biological matrices [17, 18]. Given the inherent biological variability of fecal microbiota and use of authentic, polymicrobial matrices instead of standardized monocultures, the application of the ICH M10 threshold (CV ≤ 15%) provides a scientifically appropriate benchmark. All observed CVs remained within this limit, supporting the method's suitability for quantitative viability assessment in heterogeneous bacterial suspensions such as FMT preparations.

Based on the concentration of the undiluted BioBall suspension, the validated quantification range for FMT samples extends from 1.2 × 10^8^ viable cells/mL down to the LLOQ. The manufacturer of QUANTOM Tx indicates an upper operational limit of up to 1 × 10^9^ cells/mL; however, our validation was limited by the concentration of available reference materials and focused on the range relevant for diluted FMT samples.

Robustness testing confirmed the method's reliability under standard laboratory conditions. Delays of up to two hours prior to staining did not affect cell counts (*p* > 0.05), while equivalent delays post‐staining led to a significant increase in counts (*p* < 0.05). A possible explanation is partial extracellular hydrolysis or diffusion of the dye over time, leading to increased fluorescence signals. This finding highlights the need for strict timing control, especially in the post‐staining phase, to ensure data integrity in routine QC workflows.

In contrast, quantifying *B. subtilis* spores using the Viable Cell Staining Kit proved challenging. This limitation is likely due to the intrinsic biology of endospores: limited metabolic activity (resulting in absent Calcein‐AM conversion) and the thick spore coat preventing adequate dye penetration. Consequently, the method in its current form is optimized for vegetative cells, and results regarding spore‐forming species should be interpreted with this limitation in mind. If enumeration of endospores is specifically required, complementary analytical methods such as spore germination followed by culture‐based enumeration or dedicated microscopic approaches would be necessary. Nevertheless, in the context of routine QC and dosing, reliable quantification of enzymatically active cells remains a key objective, as it provides a robust and reproducible marker for product consistency.

The Total Cell Staining Kit demonstrated good linearity for *E. coli* (1.4 × 10^7^ to 1.1 × 10^8^ cells/mL) and *B. subtilis* (9.2 × 10^6^ to 1.4 × 10^8^ cells/mL). However, a full accuracy validation was not feasible since BioBall reference materials are certified for Colony Forming Units (CFU) —representing only viable cells—rather than total cell count. Thus, while the Total Cell Staining Kit is suitable for comparative or supplementary assessments of total bacterial load, absolute quantification values should be interpreted with caution until a certified total‐count reference standard becomes available.

Since the therapeutic potential of FMT products is thought to be closely linked to the viability of the constituent microorganisms [9], robust viability assessment is essential for product development and QC. Several analytical approaches are currently available, including plate‐based CFU enumeration, PMA‐seq and flow cytometry (FACS). However, these methods are either time‐consuming, require extensive sample preparation, or demand advanced instrumentation and technical expertise, which may limit their routine use in QC environments. In this context, the QUANTOM Tx Viable Cell Staining Kit provides a pragmatic, time‐efficient, and reproducible QC‐compatible alternative by enabling rapid and standardized quantification of enzymatically active bacterial cells. By using metabolic activity as the primary readout for viability, independent of culturability, the method delivers results that are directly relevant for dosing consistency and product standardization. Its robust performance in reference monocultures and authentic, polymicrobial fecal samples supports its suitability for research, formulation development, batch release, and routine QC of microbiome‐based therapeutics. By offering simple and efficient viability quantification, this method provides valuable support for dosing verification, and regulatory compliance in FMT manufacturing.

## Author Contributions

**Maria J.G.T. Vehreschild**: supervision, conceptualization, writing – review and editing, methodology, **Timon Lang**: writing – original draft, formal analysis, visualization, investigation, methodology, conceptualization. **Fedja Farowski**: writing – review and editing, methodology, conceptualization. **Peter Langguth**: supervision, conceptualization, writing – review and editing, methodology. **Jozef Al‐gousous**: writing – review and editing, supervision, conceptualization, methodology.

## Funding

This work was supported by the German Centre for Infection Research (DZIF).

## Conflicts of Interest

The authors declare no conflicts of interest.

## Data Availability

The data that support the findings of this study are available from the corresponding author upon reasonable request.
